# Optical Coherence Tomography Angiography of Two Choroidal Nevi Variants

**DOI:** 10.1155/2017/1368581

**Published:** 2017-10-09

**Authors:** Victor M. Villegas, Armando L. Monroig, Lazaro H. Aguero, Stephen G. Schwartz

**Affiliations:** Department of Ophthalmology, Bascom Palmer Eye Institute, University of Miami Miller School of Medicine, Naples, FL, USA

## Abstract

Optical coherence tomography angiography (OCT-A) is a recently established noninvasive technology for evaluation of the retinal and choroidal vasculature. The literature regarding the findings in choroidal nevi is scarce. We report the OCT-A findings associated with two different variants. Subject one had decreased vascular flow signal in the choroidal, choriocapillaris, deep retinal, and superficial retinal layers. Subject two had decreased vascular flow signal in the choroidal, choriocapillaris, and deep retinal layers with a normal vascular flow signal in the superficial retinal layer. To our knowledge, these patterns of decreased vascular flow signals have not been previously reported using OCT-A. This may be due to blockage from the choroidal nevus, true diminished blood flow (ischemia), or other factors.

## 1. Introduction

Optical coherence tomography angiography (OCT-A) is a recently established technology that allows visualization of the chorioretinal vasculature without intravenous dye injection [1–4]. The application of this noninvasive technique is mainly designed to evaluate blood flow [1]. Various retinal and choroidal diseases have been described using OCT-A, including some intraocular tumors [2–10]. However, the literature regarding OCT-A characteristics of choroidal nevi is relatively limited [11].

The purpose of this report is to illustrate some of the OCT-A characteristics associated with two lesions diagnosed clinically as benign choroidal nevi. In both cases the commercially available Cirrus 5000 with AngioPlex (Zeiss, Jena, Germany) was used, without any subsequent image modification or processing.

## 2. Case Report

### 2.1. Subject 1

**Figure 1:**
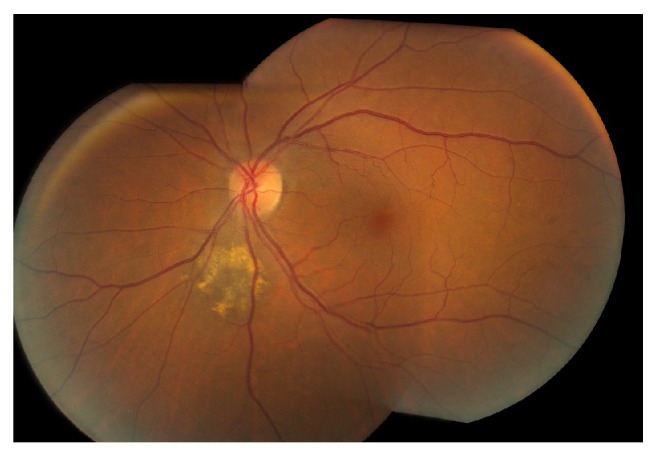
Fundus photography OS shows juxtapapillary pigmented choroidal nevus.

**Figure 2:**
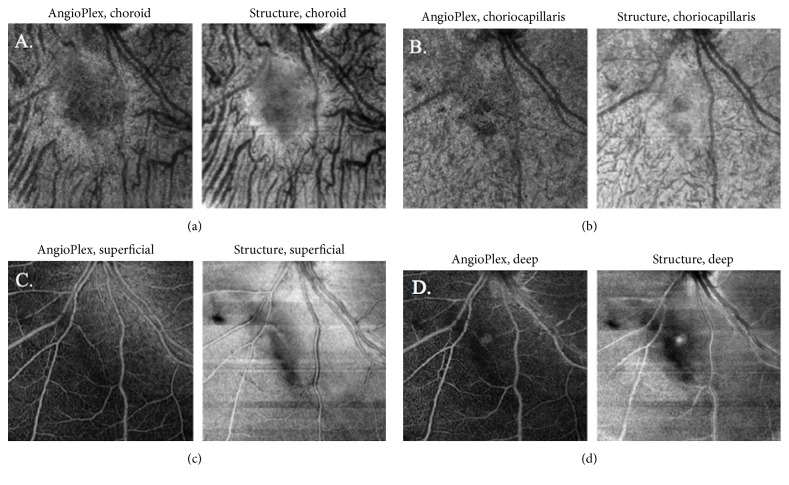
AngioPlex OCT-A 6 mm × 6 mm. (a) Choroidal plexus layer showing a decreased vascular flow signal. (b) Choriocapillary plexus layer showing a decreased vascular flow signal. (c) Superficial retinal plexus layer showing a subtle decreased vascular flow signal. (d) Deep retinal plexus layer showing a subtle decreased vascular flow signal.

A 65-year-old female with history of polymyalgia rheumatica presented for scheduled follow-up of a choroidal nevus of the left eye (OS). Visual acuity was 20/20 in each eye (OU). The examination was normal except for a juxtapapillary pigmented choroidal nevus OS, with overlying drusen, and without orange pigment or subretinal fluid (Figure 1). OCT-A 6 mm × 6 mm showed a decreased vascular flow signal in the choroidal, choriocapillaris, deep retinal, and superficial retinal layers (Figure 2).

### 2.2. Subject 2

**Figure 3:**
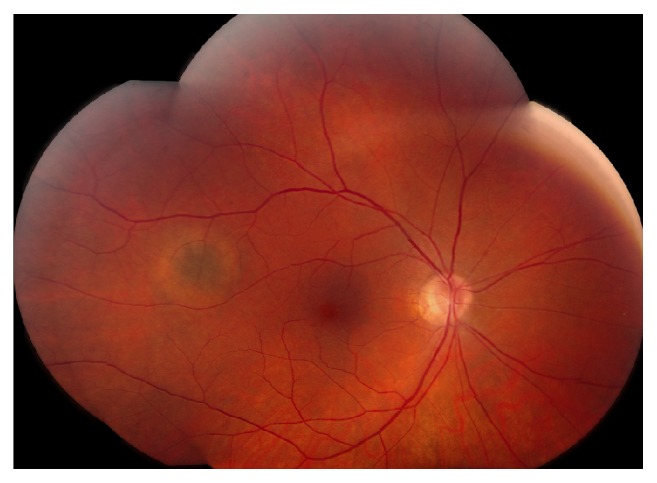
Fundus photography OD shows a temporal halo choroidal nevus.

**Figure 4:**
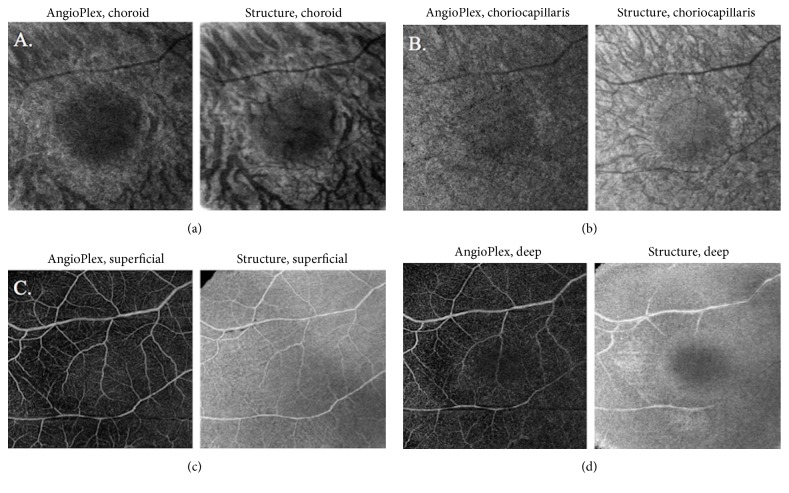
AngioPlex OCT-A 6 mm × 6 mm. (a) Choroidal plexus layer showing a decreased vascular flow signal. (b) Choriocapillary plexus layer showing a decreased vascular flow signal. (c) Superficial retinal plexus layer showing a normal vascular flow signal. (d) Deep retinal plexus layer showing a subtle central decreased vascular flow signal.

A healthy 60-year-old male presented for scheduled follow-up of a choroidal nevus of the right eye (OD). Visual acuity was 20/20 in both eyes (OU). The examination was normal except for a pigmented choroidal nevus OD with a depigmented halo around it. No drusen, orange pigment, or subretinal fluid was present (Figure 3). OCT-A 6 mm × 6 mm showed a decreased vascular flow signal in the choroidal, choriocapillaris, and deep retinal layers with a normal vascular flow signal in the superficial retinal layer (Figure 4).

## 3. Discussion

The evaluation of choroidal nevi can be a diagnostic challenge because in some cases a distinction between a benign nevus and a small choroidal melanoma is not readily apparent [12]. Multiple studies have reported hypervascularity using fluorescein angiography (FA) in patients with uveal melanoma [13, 14]. Increased vascularity in solid tumors is a hallmark of malignant transformation [15]. For example, a recent study that evaluated subjects with iris melanomas with OCT-A reported increased vascularity, with disorganized and tortuous intratumoral vascular patterns and increased vessel density [16].

OCT-A may offer certain advantages over FA in this situation. OCT-A is fast and noninvasive and has no risk of allergy. OCT-A can readily distinguish different layers of the retina and choroid, which cannot be performed with FA [17, 18]. A previous study that imaged choroidal nevi with OCT-A reported decreased vascular flow signal only in the deep retinal layer [11]. Subject 1 in the current series showed decreased vascular flow signal in the superficial, deep, and subretinal layers. Subject 2 had normal vascular flow signal in the superficial retinal plexus but decreased vascular flow signal in the deep and subretinal layers.

To our knowledge, these patterns of decreased vascular flow signals have not been previously reported using OCT-A. This may be due to blockage from the choroidal nevus, true diminished blood flow (ischemia), or other factors. If ischemia is truly present, then diminished blood flow in the superficial retina may explain the atrophic changes that are typically seen in the retina overlying some nevi [19].

OCT-A has some limitations, including expense and the relatively long image acquisition time, approximately 3 seconds. Some patients cannot hold their gaze for this long, especially because most nevi are not in the macula. Furthermore, inadequate penetration through highly pigmented or thick tumors may limit the use of this technology to small nevi/melanomas (less than 2 mm in thickness). It might be interesting to compare OCT, OCT-A, and B-scan but echography was not performed on these two patients because the lesions were clinically flat.

In summary, OCT-A may provide a simple, quick, and safe way to monitor choroidal nevi. OCT-A may be particularly useful in cases with high-risk features to detect changes in vascularity. As more clinical examples are collected, our understanding of using OCT-A to image pigmented lesions should increase.
